# Spirit of the Medical Press

**Published:** 1850-07

**Authors:** 


### Part 3.---Abstracts.

### ARTICLE I.

### SPIRIT OF THE MEDICAL PRESS.

*Practical Medicine.*---In the May No. of the N. O. Medical and Surgical Journal is a judicious paper on the subject of Typhoid Fever, by Dr. J. C. Harris, of Wetumpka, Ala. He contends that the true Typhoid Fever, of authors, is very seldom seen by American practitioners of the South and West, and that the disease which has been erroneously so called, is nothing more than a continued Malarial Fever. His remarks are very pointed, and we extract his closing remark, as worthy the consideration of those attacked with the mania for getting their medical opinions from Europe.

"In now bringing to a close our remarks, and dismissing, for the present, the further consideration of the subject of Typhoid Fever, we venture the prediction, that had Dr. Bartlett, instead of visiting the hospitals on the eastern continent, and studying there the fevers of the Mississippi valley and the South generally, spent some time in observing and treating them in the malarious localities where they are engendered and prevail, the profession would, probably, never have been favored with his decidedly learned and entirely elegant treatise on Typhoid Fever; and it still remains, to some extent, we think, a matter of regret, that since this publication, so many Southern physicians, like an ancient philosopher on another occasion, with this talismanic book in one hand, and a lighted taper in the other, have gone forth at the *summer solstice*, at mid-day, under a burning sun, and amidst scorching sands and malarious hot-beds, in search of this, to them, until then, *great unknown*, and that, after many a weary pilgrimage, some, more fortunate than others, have returned crying (*Eureka*), I have found it. To all of these we tender our most profound acknowledgments and congratulations, with an assurance that we really, in viewing them in the light of discoverers, feel happy in the contemplation of the glorious felicity that awaits and surrounds them."

In the Boston Journal, for May 1st, is an interesting paper by Dr. Ware, on Croup. The author advocates a mode of treatment which he conceives to be more rational than the method heretofore pursued. He thinks emetics often prove injurious. His mode of treatment consists in the use of Calomel and Opium, and the local application of Nitrate of Silver. We doubt whether there are many of our readers who will agree with Dr. Ware; believing that the administration of emetics is founded on sound views of the pathology of the disease. Dr. W.'s notices of the pathology of Croup, are very interesting. The principal point is embodied in the following extract:

"Now when we examine the cases of recovery of membranous Croup which actually take place, and compare them with the condition of the parts in those which are examined after death, we find very clear evidences of a tendency in the disease to go through a certain course of changes which will terminate in health. The false membrane is effused, and, at the same time, the mucous membrane is thickened and congested. After a time, a process of suppuration is established upon the surface of the mucous membrane, underneath the false membrane, which of course separates the latter from the former, so that it lies loosely upon it, whilst between them is a layer of pus. If the membrane thus thrown off be thick and strong, it is expectorated in distinct pieces, sometimes of a considerable size; if it be thin and less firm, it is either converted partially into pus, or else is broken up into smaller shreds, and mixed with the pus so as not to be distinguished from it, except by very careful examination, and thus it is all gradually thrown up. The diseased membrane does not free itself from the false membrane over its whole surface at once. Those portions from which the false membrane has separated are left in an inflamed and irritable state; the expectorated membrane and pus are often tinged with blood, probably from the fact that by the violent effort of coughing, some portions are torn off from the mucous surface before the purulent process has effected a complete separation. The cough, then, with more or less expectoration, and a hoarseness, in some cases amounting to an incapacity for speaking except in a whisper, continue for some time—the affection of the voice for several weeks. The parts are at length, however, perfectly restored."

In No. XIX. of Braithwaite's Retrospect is an interesting account, by Mr. Hannon, on the use of Manganese in Anœmic, and other affections. Several cases are detailed, in which its use was beneficial.

[" Several illustrative cases are given. The first mentioned is one of extreme chlorosis, in which the patient was sent into country air, and took iron for some time, without benefit. We are told that]

" The patient was then directed to take one of the following pills daily before dinner: Extract of Cinchona, Carbonate of Manganese, of each a drachm. Mix and divide into four grain pills. After she had used these pills for a fortnight, the cheeks and conjunctivæ regained their colour, and the swelling of the feet disappeared. The following pills were then ordered: Sulphate of Manganese, Carbonate of Soda, of each a drachm; fresh charcoal and honey, of each a sufficient quantity to make a mass, to be divided into four-grain pills. A fortnight after the employment of this medicine, the bellows-sound had disappeared; the pulsations of the heart were strong and loud; and an energetic impulse was felt on applying the hand. There was no syncope, and the appetite had returned. The dose of the pills was increased; and a month after, menstruation occurred, and the patient became plump, and able to bear much exertion. She digested and slept well---in a word, was cured.

["Another case was that of a young lady affected with phthisis:]

" Iron, with opium was prescribed; but it increased the cough, and brought on obstinate constipation. Syrup of the Phosphate of Manganese was then given, with Cod-liver Oil; the latter being added rather to prevent the contact of air with the Manganese, than from any expectation of its prodncing good effects. The constipation ceased; and the cough became more bearable, and ceased in a fortnight. The patient then began to recover *embonpoint.* A month after the knuckles assumed a very remarkable brick-red color, which has continued up to the present time---a period of nearly a year and a-half. This patient took three *gros* (216 grains) of Phosphate of Manganese, in doses of three grains daily.

"Madame R. was affected with a cancer of the uterus. She complained of remittent pain in the hypogastric region, and suffered much while at stool. In the evening, she was troubled with severe lancinating pains, which often continued through the night. She was excessively weak, and of a pale yellow hue. She was troubled with palpitation, and a *bruit* was heard in the carotid. The feet frequently swelled. Syrup of the Iodide of Manganese was given with syrup of Horse-Radish, for several months. The pains did not leave her, but the anæmic appearance completely disappeared. To calm the pains, Opium, with extract of Hemlock, was prescribed; and the patient became apparently cured.

"Mademoiselle M., aged 14, of a scrofulous constitution, had glandular enlargements in the neck. ulceration of the transparent cornea of the left eye, and caries of the first phalangeal bone of the index finger of the right hand. Being the daughter of a peasant, she had lived exclusively on vegetable food; but was ordered to take meat and to drink beer. Syrup of the Iodide of Manganese was given in doses of a spoonful two or three times a-day. Under the influence of this, and her improved diet, she became less lean; soon after, the cornea regained its transparency, having been washed with a lotion containing gr. ss. of Nitrate of Silver, to an ounce of Distilled Water. The suppuration of the carious bone ceased, and the finger was cured.

M. G. B., aged 38, had been treated with mercury for some years, for constitutional Syphilis. The bones were sound; the skin was affected with all kinds of eruption; the tongue had long been the seat of an obstinate tumour; and there were Syphilitic Opthalmia and iritis. Fumigation and Iodide of Potassium were persevered in for several months, but without effect. Iodide of Manganese was then given, with syrup of Sarsaparilla; and in a month the patient was completely healed. He was directed to continue the use of the Maganese; and, as he has not since applied for relief, it is probable that he has had no relapse."

In the same Number is an account of the use of Coffee in Hooping-Cough. Should the remedy accomplish what is claimed for it, Dr. Guyot will prove to have made a great discovery.

" A child of four years old, under rigid dietetic treatment for measles, was seized with congestion of the lungs, against which energetic antiphlogistic treatment was put in force. When Dr. Guyot was called in, death seemed imminent from violent paroxysms of cough, which induced such alarming suffocation and syncope, as to cause the patient to appear to be actually dead. Some spoonfuls of strong beef-tea, with some spoonfuls of sweet and hot infusions of the lime-tree, were prescribed. The child passed the night without fainting. On the following day, the same infusions were continued, and in addition, there was given a little bit of grilled and finely minced mutton, with the view of restoring strength as rapidly as possible: but a severe fit of coughing came on, which, terminating in vomiting, caused ejection of the food; recourse was again had to the beef-tea, which provoked a similar fit of coughing, and was ejected. In these circumstances, with death apparently impending, a tea-spoonful of coffee was given after each tea-spoonful of beef-tea. Not only was the beef-tea retained, but, to my great surprise, the cough likewise ceased. Two hours afterwards, the child took, with relish, a little bit of hashed cutlet, followed by a a tea-spoonful of the coffee; the food was not ejected, digestion went on naturally, the night was passed in comfortable sleep, there was no more cough:—and in truth, the child was saved. On the following day, the treatment was continued, and there was a continuance of a like satisfactory state. On the third day, however, the relations omitted the coffee, when the cough returned after the first meal, with all the characterestic violence of well-formed Hooping-Cough; and this state continued during the day. On the fourth day, when the coffee was resumed, the cough disappeared. During forty-seven days the coffee was persisted in, when the cure was distinctly complete. The little patient was the only daughter of M. Haquin, a master boot-maker of Argenteuil.

" This case was furnishingsubject of serious reflection, when Dr. Guyot met, in a public conveyance, M. Bouja, ex-notary of Franconville. He told him that he had been obliged to have double doors for his study, to keep out the terible and incessant noise caused by his two children with Hooping-Cough.

" Dr. Guyot detailed the case above narrated, and he cured both his children in four days by means of coffee. He has since tried the remedy successfully in above sixty cases. The efficacy of coffee in Hooping-Cough seems to show that the seat of the disease is not in the bronchial tubes or larynx, nor in their vascular or nervous network, but exclusively in the digestive organs, and especially in the pharynx; which, again, probably depends on some special affection of the stomach."

### TREATMENT OF REMITTENT FEVERS.

We observe that our friends of the eastern cities are beginning to believe the doctrines so generally taught, and to adopt the treatment so successfully practiced in the West and South, in Remittent Fevers.

At a late meeting of the Philadelphia County Medical Society, Dr. Isaac Parish, an able and distinguished practitioner of the city, remarked:—

"It is well known that, within a few years past, the practice of administering Quinine in the early stage of Malarial Fevers of the remittent type, has become quite prevalent in those sections of the United States where these fevers are most prevalent. The idea is now extensively entertained, that Remittent and Intermittent Fevers are essentially the same disease, and that the absence of intermissions in the former, should not prevent the administration of the remedy which is found so effectual in controlling the latter. Hence, the physicians who hold to this opinion, give Quinine in large doses, and with a view to its anti-periodic qualities, in the remissions of a Fever, for the purpose of preventing the exacerbrations, which are known to occur at particular intervals. They do not wait until all febrile action has passed away, or until a crisis has occurred in the natural course of the Fever, on the fifth, seventh, or ninth day, or even later, but they commence at the outset with this potent remedy, just as in the early stage of a remittent, after a free evacuation of the bowels by a mercurial cathartic, and when a distinct morning remission was observable, has had the effect of moderating the evening exacerbation, and by continuing it a few days, of bringing the Fever to a rapid and favorable crisis. I have seldom given over 15 or 20 grains in the remission, and in mild cases, have found even less than this to answer the purpose. Since adopting this plan I have they would do if no Fever were present, as in the intermission of an intermittent. In both cases, they would, perhaps, generally precede the Quinine by a brisk mercurial cathartic, and then attack it with Quinine, in the interval of the paroxysms, with a view to cutting short the disease.

The results of this practice, as reported to us by many highly respectable physicians of the West and South, whose experience has been ample, is, that remittent fevers which, under the plan of treatment formerly in vogue, were accustomed to run a protracted course, and to assume a serious aspect, are brought to a rapid termination, or converted, after two or three paroxysms, into the intermittent type, and easily cured. They say, that so far from the Quinine increasing the heat, thirst, and sensorial disturbances incident to a fever, that it allays these, calms the nervous system, and acts as a diaphoretic. Now, it is a point of great practical interest, to know how far these observations of our brethren in other sections, correspond with experience here ; and my object in introducing the subject at this time is to ascertain this.

For myself, I can state that, in common with most of those who hear me, I was educated in the doctrine, that in remittents, we must be very cautious of bark or Quinine, while the least febrile excitement is present.

The idea entertained here, by the most eminent medical teachers, when I was a student, was, that in remittent, the febrile excitement must be reduced by the lancet and brisk purgation during the early stages, and that tonics were only to be given after the decline of the fever. Even my excellent father, who, as is well known by many here, was more favorable to the use of tonics and supporting treatment in Fever than most of his contemporaries, taught in his lectures, that bark and Quinine were not to be given until the cleaning of the tongue, or the occurrence of critical sweats, indicated the approach of convalescence ; except in cases where malignant symptons were developed, or where unusual evidence of prostration existed,—and then the article was given more for its general tonic properties, than with any prospect of cutting short the fever.

Within the past few years, however, from the large amount of testimony to the value of Quinine in the early stages of remittent, which has reached us through the medical journals, I have been induced to pursue this course, and have reason to be well satisfied with it. I have found that Quinine, given not seen any cases of protracted remittent, running on for three or four weeks, and attended with tympanitis, subsultus tendinum, low delirium, hemorrhage from the bowels, &c. Whether this fortunate exemption is attributable to the mode of treatment adopted, or to a diminished liability in our Fevers of late years to assume this form, I cannot positively say ; though I am inclined think that the old plan of delaying the use of tonics, may have contributed to the production of these prolonged cases.

If the practice recently introduced by the practitioners of the South and West, in the treatment of Malarial Fevers, be confirmed by experience, and established as sound, it must be admitted that a great step has been taken in practical medicine ; diseases, once the dread of the inhabitants of large sections of our country, will be divested of much of their virulence, and be brought under the control of remedial measures, while on science will have achieved a grand triumph.—*Med. Examiner.*

*Surgery.*---An interesting case has occurred in the hands of Prof. Hamilton, of Buffalo. A woman lost a phalanx of her thumb, from necrosis. Prof. H. applied a roller, and had the satisfaction of seeing a new phalanx formed. This, though a frequent occurrence in the crustacea tribe, is certainly very rare in the human being. M.

